# 9-(3,4-Dimeth­oxy­phen­yl)-3,3,6,6-tetra­methyl-1,2,3,4,5,6,7,8,9,10-deca­hydro­acridine-1,8-dione

**DOI:** 10.1107/S1600536813002250

**Published:** 2013-01-26

**Authors:** Rajni Kant, Vivek K. Gupta, Kamini Kapoor, D. R. Patil, D. R. Chandam, Madhukar B. Deshmukh

**Affiliations:** a X-ray Crystallography Laboratory, Post-Graduate Department of Physics & Electronics, University of Jammu, Jammu Tawi 180 006, India; b Department of Chemistry, Shivaji University, Kolhapur, 416 004 (MS), India

## Abstract

The asymmetric unit of the title compound, C_25_H_31_NO_4_, contains two independent mol­ecules. In one mol­ecule, the benzene ring and an attached meth­oxy group were refined as disordered over two sets of sites in a 0.65 (4): 0.35 (4) ratio. In both mol­ecules, the central ring of the acridinedione system adopts a flattened boat conformation. The four essentially planar atoms of this ring [maximum deviations = 0.006 (5) Å in both mol­ecules] forms dihedral angles of 86.8 (2) and 87.6 (2)°, respectively, with the major and minor components in the disordered benzene ring and 87.3 (2)° with the benzene ring in the fully ordered mol­ecule. The two outer rings of the acridinedione system adopt sofa conformations in both mol­ecules. In the crystal, N—H⋯O hydrogen bonds form two independent chains along [100]. C—H⋯O hydrogen bonds link the chains, forming a three-dimensional network.

## Related literature

For applications of acridines, see: Murugan *et al.* (1998 ▶); Leon *et al.* (2008 ▶); Josephrajan *et al.* (2005 ▶); Srividya *et al.* (1998 ▶, 1996 ▶). For related structures, see: Balamurugan *et al.* (2009 ▶); Zhao & Teng (2008 ▶); Kant *et al.* (2013*a* ▶,*b* ▶). For ring conformations, see: Duax & Norton (1975 ▶).
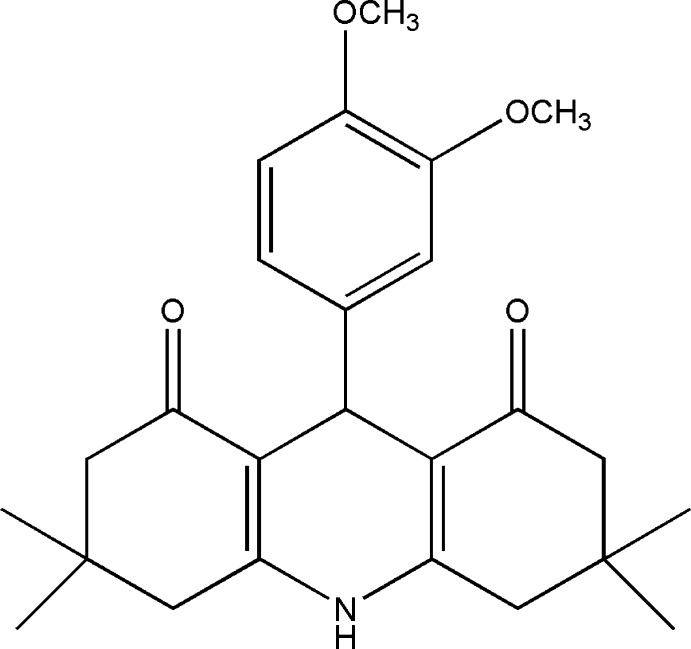


## Experimental

### 

#### Crystal data

C_25_H_31_NO_4_
*M* *_r_* = 409.51
Orthorhombic, 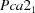

*a* = 14.1607 (6) Å
*b* = 15.3126 (10) Å
*c* = 21.1196 (14) Å
*V* = 4579.5 (5) Å^3^
*Z* = 8
Mo *K*α radiation
μ = 0.08 mm^−1^
*T* = 293 K
0.3 × 0.2 × 0.2 mm

#### Data collection

Oxford Diffraction Xcalibur Sapphire3 diffractometer
Absorption correction: multi-scan (*CrysAlis PRO*; Oxford Diffraction, 2010 ▶) *T*_min_ = 0.672, *T*_max_ = 1.000
14639 measured reflections
6386 independent reflections
4075 reflections with *I* > 2σ(*I*)
*R*_int_ = 0.055

#### Refinement

*R*[*F* ^2^ > 2σ(*F* ^2^)] = 0.059
*wR*(*F* ^2^) = 0.127
*S* = 1.01
6386 reflections
604 parameters
53 restraints
H-atom parameters constrained
Δρ_max_ = 0.17 e Å^−3^
Δρ_min_ = −0.17 e Å^−3^

### 

Data collection: *CrysAlis PRO* (Oxford Diffraction, 2010 ▶); cell refinement: *CrysAlis PRO*; data reduction: *CrysAlis PRO*; program(s) used to solve structure: *SHELXS97* (Sheldrick, 2008 ▶); program(s) used to refine structure: *SHELXL97* (Sheldrick, 2008 ▶); molecular graphics: *ORTEP-3 for Windows* (Farrugia, 2012 ▶) and *PLATON* (Spek, 2009 ▶); software used to prepare material for publication: *PLATON*.

## Supplementary Material

Crystal structure: contains datablock(s) I, New_Global_Publ_Block. DOI: 10.1107/S1600536813002250/lh5574sup1.cif

Structure factors: contains datablock(s) I. DOI: 10.1107/S1600536813002250/lh5574Isup2.hkl

Supplementary material file. DOI: 10.1107/S1600536813002250/lh5574Isup3.cml

Additional supplementary materials:  crystallographic information; 3D view; checkCIF report

## Figures and Tables

**Table 1:** Hydrogen-bond geometry (Å, °)

*D*—H⋯*A*	*D*—H	H⋯*A*	*D*⋯*A*	*D*—H⋯*A*
N12*A*—H12*A*⋯O1*A* ^i^	0.86	2.07	2.846 (4)	150
N12*B*—H12*B*⋯O8*B* ^ii^	0.86	2.05	2.885 (4)	165
C2*A*—H2*AA*⋯O1*B* ^ii^	0.97	2.45	3.400 (6)	166
C16*A*—H16*D*⋯O25*A* ^iii^	0.96	2.61	3.477 (12)	150
C7*A*—H7*AB*⋯O27*B* ^iv^	0.97	2.47	3.432 (6)	169
C7*B*—H7*BB*⋯O8*A* ^i^	0.97	2.57	3.507 (5)	162

Symmetry codes: (i) 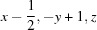; (ii) 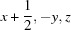; (iii) 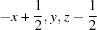; (iv) 
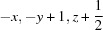
.

**Fig. 1.**
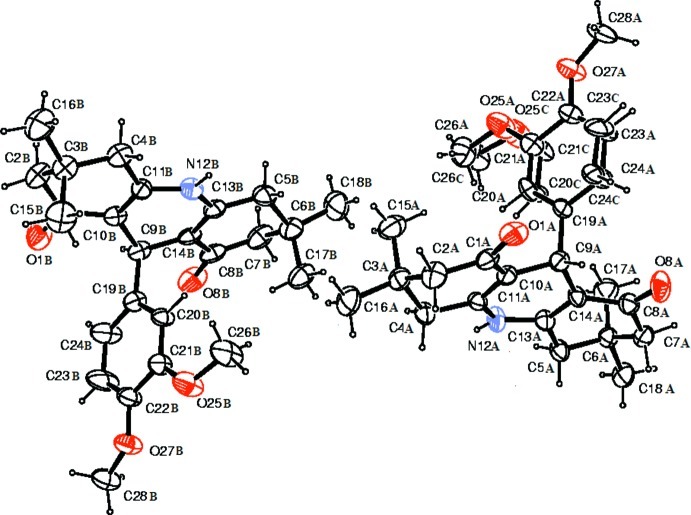
The asymmetric unit of the title compound with ellipsoids drawn at the 40% probability level. H atoms are shown as small spheres of arbitrary radii. Atoms labeled with 'C' are the minor component of disorder.

**Fig. 2.**
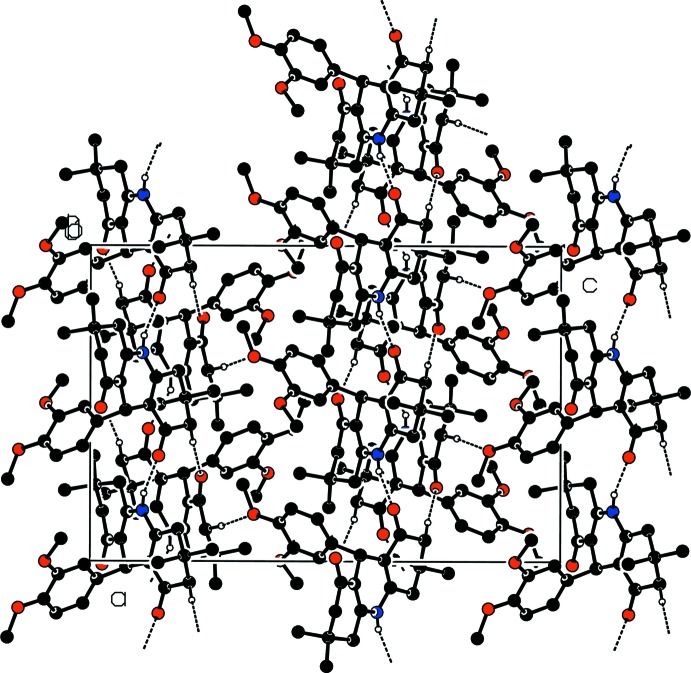
The packing arrangement of molecules viewed along the b axis. The dashed lines show intermolecular C—H···O and N—H···O hydrogen bonds. Only H atoms involved in hydrogen bonds are shown. The disorder is not shown.

## supplementary crystallographic information

### Comment

The 1,4- dihydropyridine (DHP) nucleus act as a versatile intermediate for the synthesis of several pharmaceuticals together with those of cardiovascular drugs and as a calcium channel modulators, laser dyes and photo initiators (Leon *et al.*, 2008). Acridines, the earliest known antibiotics and are toxic towards bacteria. Some acridinedione derivatives show good inhibition against the pathogen Vibrio isolate-I (Josephrajan *et al.*, 2005). Certain acridine-1,8-diones exhibit fluorescence activities (Murugan *et al.*, 1998) and a few acridinedione derivatives also show photophysical (Srividya *et al.*, 1998) and electrochemical properties (Srividya *et al.*, 1996). Thus, the accurate description of crystal structures of substituted acridinediones are expected to provide useful information on the role of substituents in influencing molecular conformation which has a direct relationship to biological activity. This paper deals with the crystal structure of a 3,4-dimethoxyphenyl substituted tetramethyl acridinedione, (I).

The asymmetric unit of the title compound comprises of two crystallographically independent molecules, A and B (Fig.1). In molecule A, the benzene ring and one attached methoxy group is disordered over two sets of sites in a 0.65 (4):0.35 (4) ratio. Bond lengths and angles are normal and correspond to those observed in related structures (Balamurugan *et al.*, 2009; Zhao & Teng 2008; Kant *et al.* (2013*a*,*b*)). The central ring (C9/C10/C11/N12/C13/C14) of the acridinedione moiety adopts a flattened boat conformation (ΔCs(C9A) = 1.32 & ΔCs (C13A—C14A) = 10.43; ΔCs(C9B) = 1.34 & ΔCs (C13B—C14B) = 12.58) and the four essentially planar atoms (C10/C11/C13/C14) of this ring (maximum deviation-0.006 (5) Å for C11A and -0.006 (5) Å for C13B) forms a dihedral angle of 86.8 (2)° and 87.6 (2)° with the major and minor components of disorder benzene ring in molecule A and 87.3 (2)° with the benzene ring in molecule B. Both the outer rings adopt sofa conformations (ΔCs (C3A) = 7.99; ΔCs (C6A) = 4.61; ΔCs (C3B) = 8.34; ΔCs (C6B) = 8.93) (Duax & Norton, 1975). In the crystal N—H···O hydrogen bonds (Table 1) form two independent one-dimensional chains parallel to [100]. In addition weak C—H···O hydrogen bonds link chains to form a three-dimensional network (Fig. 2).

### Experimental

In a 50 ml round bottom flask, a mixture of dimedone (2 mmole), 3,4 - dimethoxy benzaldehyde 2(1 mmole) and ammonium acetate (1.2 mmole) in aqu. ethanol (7 ml) was stirred at room temperature for 5 min. To this [CMIM][HSO4](3-carboxymethyl-1-methylimidazolium bisulfate)(20 mol %) was added and the reaction mixture heated at 348–351 K for 1.5 hrs. The progress of reaction was monitored by TLC. After completion of reaction, the mixture was gradually cooled to RT and poured on ice water under stirring. A precipitate was formed which was filtered and dried. The crude product was recrystallized from ethanol.

M.P.: 559–563 K, Yield:78%. IR(KBr): 3273, 3200, 3071, 2954, 1642, 1610 cm-1. 1H NMR(300 MHz, DMSO-d6): δ = 7.9 (s, 1H, NH); 7.2–6.2 (m, 3H, Ar—H); 5.0 (s, 1H, CH); 3.8 (s, 3H, OCH3); 3.7 (s, 3H, OCH3); 2.3–2.1 (m, 8H, CH2); 1.0 (s, 6H, CH3); 0.9 (s,6*H*, CH3).

### Refinement

All H atoms were positioned geometrically and were treated as riding on their parent C atoms, with N—H distances of 0.86 Å, C—H distances of 0.93–0.98 Å and with *U*_iso_(H) = 1.2*U*_eq_(C/N) or 1.5*U*_eq_(methyl C).

### Crystal data

**Table:**

C_25_H_31_NO_4_	*F*(000) = 1760
*M**_r_* = 409.51	*D*_x_ = 1.188 Mg m^−^^3^
Orthorhombic, *P**c**a*2_1_	Mo *K*α radiation, λ = 0.71073 Å
Hall symbol: P 2c -2ac	Cell parameters from 6069 reflections
*a* = 14.1607 (6) Å	θ = 3.5–29.0°
*b* = 15.3126 (10) Å	µ = 0.08 mm^−^^1^
*c* = 21.1196 (14) Å	*T* = 293 K
*V* = 4579.5 (5) Å^3^	Block, yellow
*Z* = 8	0.3 × 0.2 × 0.2 mm

### Data collection

**Table:**

Oxford Diffraction Xcalibur Sapphire3 diffractometer	6386 independent reflections
Radiation source: fine-focus sealed tube	4075 reflections with *I* > 2σ(*I*)
Graphite monochromator	*R*_int_ = 0.055
Detector resolution: 16.1049 pixels mm^-1^	θ_max_ = 25.0°, θ_min_ = 3.5°
ω scans	*h* = −16→16
Absorption correction: multi-scan (*CrysAlis PRO*; Oxford Diffraction, 2010)	*k* = −18→13
*T*_min_ = 0.672, *T*_max_ = 1.000	*l* = −25→21
14639 measured reflections	

### Refinement

**Table:**

Refinement on *F*^2^	Primary atom site location: structure-invariant direct methods
Least-squares matrix: full	Secondary atom site location: difference Fourier map
*R*[*F*^2^ > 2σ(*F*^2^)] = 0.059	Hydrogen site location: inferred from neighbouring sites
*wR*(*F*^2^) = 0.127	H-atom parameters constrained
*S* = 1.01	*w* = 1/[σ^2^(*F*_o_^2^) + (0.0377*P*)^2^] where *P* = (*F*_o_^2^ + 2*F*_c_^2^)/3
6386 reflections	(Δ/σ)_max_ = 0.002
604 parameters	Δρ_max_ = 0.17 e Å^−^^3^
53 restraints	Δρ_min_ = −0.17 e Å^−^^3^

### Special details

**Table:**

Experimental. *CrysAlis PRO*, Oxford Diffraction Ltd., Version 1.171.34.40 (release 27–08-2010 CrysAlis171. NET) (compiled Aug 27 2010,11:50:40) Empirical absorption correction using spherical harmonics, implemented in SCALE3 ABSPACK scaling algorithm.
Geometry. All e.s.d.'s (except the e.s.d. in the dihedral angle between two l.s. planes) are estimated using the full covariance matrix. The cell e.s.d.'s are taken into account individually in the estimation of e.s.d.'s in distances, angles and torsion angles; correlations between e.s.d.'s in cell parameters are only used when they are defined by crystal symmetry. An approximate (isotropic) treatment of cell e.s.d.'s is used for estimating e.s.d.'s involving l.s. planes.
Refinement. Refinement of *F*^2^ against ALL reflections. The weighted *R*-factor *wR* and goodness of fit *S* are based on *F*^2^, conventional *R*-factors *R* are based on *F*, with *F* set to zero for negative *F*^2^. The threshold expression of *F*^2^ > σ(*F*^2^) is used only for calculating *R*-factors(gt) *etc*. and is not relevant to the choice of reflections for refinement. *R*-factors based on *F*^2^ are statistically about twice as large as those based on *F*, and *R*- factors based on ALL data will be even larger.

### Fractional atomic coordinates and isotropic or equivalent isotropic displacement parameters (Å^2^)

**Table:**

	*x*	*y*	*z*	*U*_iso_*/*U*_eq_	Occ. (<1)
O1A	0.41575 (18)	0.5230 (2)	0.62071 (19)	0.0548 (9)	
O8A	0.26645 (19)	0.7813 (2)	0.73491 (19)	0.0666 (10)	
O27A	0.4145 (2)	0.4704 (2)	0.92959 (18)	0.0681 (10)	
N12A	0.0923 (2)	0.5381 (2)	0.6676 (2)	0.0474 (10)	
H12A	0.0388	0.5115	0.6674	0.057*	
C1A	0.3348 (3)	0.4965 (3)	0.6129 (3)	0.0428 (12)	
C2A	0.3141 (3)	0.4280 (3)	0.5643 (3)	0.0550 (13)	
H2AA	0.3702	0.3927	0.5582	0.066*	
H2AB	0.3000	0.4565	0.5244	0.066*	
C3A	0.2315 (3)	0.3681 (3)	0.5818 (2)	0.0458 (11)	
C4A	0.1465 (2)	0.4260 (3)	0.5952 (2)	0.0448 (11)	
H4AA	0.1228	0.4491	0.5555	0.054*	
H4AB	0.0969	0.3909	0.6140	0.054*	
C5A	0.0075 (3)	0.6611 (3)	0.7126 (2)	0.0445 (11)	
H5AA	−0.0369	0.6173	0.7272	0.053*	
H5AB	−0.0182	0.6874	0.6745	0.053*	
C6A	0.0183 (3)	0.7312 (3)	0.7634 (2)	0.0412 (10)	
C7A	0.0994 (3)	0.7905 (3)	0.7444 (2)	0.0465 (12)	
H7AA	0.0810	0.8232	0.7071	0.056*	
H7AB	0.1104	0.8322	0.7782	0.056*	
C8A	0.1907 (3)	0.7431 (3)	0.7306 (2)	0.0439 (11)	
C9A	0.2752 (2)	0.6035 (3)	0.6965 (2)	0.0407 (11)	
H9AA	0.3221	0.6436	0.6786	0.049*	
C10A	0.2565 (2)	0.5319 (3)	0.6482 (2)	0.0369 (10)	
C11A	0.1688 (3)	0.5000 (3)	0.6383 (2)	0.0351 (11)	
C13A	0.0997 (2)	0.6177 (3)	0.6969 (2)	0.0382 (10)	
C14A	0.1847 (2)	0.6543 (3)	0.7080 (2)	0.0365 (10)	
C15A	0.2574 (3)	0.3134 (3)	0.6400 (3)	0.0685 (16)	
H15D	0.2033	0.2805	0.6534	0.103*	
H15E	0.2773	0.3514	0.6736	0.103*	
H15F	0.3078	0.2742	0.6294	0.103*	
C16A	0.2084 (3)	0.3074 (3)	0.5267 (3)	0.0715 (16)	
H16D	0.1920	0.3416	0.4902	0.107*	
H16E	0.1562	0.2706	0.5380	0.107*	
H16F	0.2625	0.2719	0.5172	0.107*	
C17A	0.0379 (3)	0.6895 (3)	0.8280 (2)	0.0603 (13)	
H17D	0.0908	0.6506	0.8245	0.090*	
H17E	−0.0167	0.6574	0.8415	0.090*	
H17F	0.0519	0.7344	0.8583	0.090*	
C18A	−0.0729 (3)	0.7854 (3)	0.7663 (3)	0.0587 (13)	
H18D	−0.0866	0.8084	0.7250	0.088*	
H18E	−0.0649	0.8327	0.7956	0.088*	
H18F	−0.1241	0.7490	0.7799	0.088*	
C19A	0.3146 (3)	0.5673 (3)	0.7580 (2)	0.0402 (10)	
C22A	0.3848 (3)	0.5054 (3)	0.8740 (2)	0.0494 (12)	
O25A	0.2757 (9)	0.3913 (8)	0.8741 (5)	0.074 (4)	0.65 (4)
C20A	0.2766 (6)	0.4936 (5)	0.7872 (3)	0.043 (3)	0.65 (4)
H20B	0.2269	0.4644	0.7676	0.052*	0.65 (4)
C21A	0.3099 (6)	0.4630 (5)	0.8431 (3)	0.045 (2)	0.65 (4)
C23A	0.4224 (6)	0.5783 (6)	0.8451 (4)	0.065 (4)	0.65 (4)
H23B	0.4721	0.6075	0.8647	0.078*	0.65 (4)
C24A	0.3881 (6)	0.6096 (6)	0.7876 (4)	0.055 (3)	0.65 (4)
H24B	0.4148	0.6589	0.7692	0.066*	0.65 (4)
C26A	0.2016 (14)	0.3452 (12)	0.8480 (7)	0.078 (6)	0.65 (4)
H26D	0.1777	0.3044	0.8786	0.117*	0.65 (4)
H26E	0.2231	0.3141	0.8113	0.117*	0.65 (4)
H26F	0.1523	0.3849	0.8361	0.117*	0.65 (4)
O25C	0.2505 (17)	0.420 (3)	0.8889 (11)	0.092 (9)	0.35 (4)
C20C	0.2630 (12)	0.5092 (18)	0.7952 (8)	0.043 (4)	0.35 (4)
H20C	0.2046	0.4898	0.7808	0.052*	0.35 (4)
C21C	0.2953 (12)	0.4801 (19)	0.8519 (8)	0.056 (5)	0.35 (4)
C23C	0.4336 (12)	0.5662 (16)	0.8386 (9)	0.062 (5)	0.35 (4)
H23C	0.4891	0.5898	0.8549	0.075*	0.35 (4)
C24C	0.4021 (14)	0.593 (2)	0.7793 (10)	0.055 (3)	0.35 (4)
H24C	0.4401	0.6286	0.7541	0.066*	0.35 (4)
C26C	0.184 (3)	0.365 (3)	0.8620 (15)	0.081 (14)	0.35 (4)
H26G	0.1587	0.3272	0.8939	0.122*	0.35 (4)
H26H	0.2137	0.3307	0.8295	0.122*	0.35 (4)
H26I	0.1344	0.3993	0.8438	0.122*	0.35 (4)
C28A	0.5051 (4)	0.4963 (4)	0.9522 (3)	0.0720 (18)	
H28D	0.5200	0.4642	0.9899	0.108*	
H28E	0.5045	0.5577	0.9615	0.108*	
H28F	0.5518	0.4846	0.9204	0.108*	
O1B	−0.0121 (2)	−0.2853 (2)	0.5241 (2)	0.0810 (12)	
O8B	−0.16622 (19)	−0.0344 (2)	0.6456 (2)	0.0734 (12)	
O25B	−0.0012 (2)	0.1058 (2)	0.40468 (18)	0.0766 (11)	
O27B	−0.1489 (2)	0.0429 (2)	0.34795 (17)	0.0604 (9)	
N12B	0.1604 (2)	−0.0524 (2)	0.61100 (19)	0.0458 (10)	
H12B	0.2141	−0.0265	0.6140	0.055*	
C1B	0.0648 (3)	−0.2511 (3)	0.5358 (3)	0.0541 (13)	
C2B	0.1562 (3)	−0.2996 (3)	0.5243 (3)	0.0589 (14)	
H2BA	0.1472	−0.3398	0.4893	0.071*	
H2BB	0.1710	−0.3340	0.5616	0.071*	
C3B	0.2397 (3)	−0.2402 (3)	0.5095 (2)	0.0506 (12)	
C4B	0.2483 (3)	−0.1713 (3)	0.5617 (2)	0.0502 (12)	
H4BA	0.2742	−0.1983	0.5995	0.060*	
H4BB	0.2920	−0.1263	0.5481	0.060*	
C5B	0.1020 (3)	0.0532 (3)	0.6868 (3)	0.0531 (13)	
H5BA	0.1536	0.0892	0.6714	0.064*	
H5BB	0.1228	0.0258	0.7258	0.064*	
C6B	0.0174 (3)	0.1123 (3)	0.7014 (3)	0.0533 (13)	
C7B	−0.0673 (3)	0.0519 (3)	0.7119 (3)	0.0622 (14)	
H7BA	−0.0574	0.0193	0.7507	0.075*	
H7BB	−0.1233	0.0875	0.7177	0.075*	
C8B	−0.0852 (3)	−0.0110 (3)	0.6595 (3)	0.0491 (14)	
C9B	−0.0208 (2)	−0.1138 (3)	0.5758 (2)	0.0424 (11)	
H9BA	−0.0683	−0.1550	0.5913	0.051*	
C10B	0.0698 (3)	−0.1646 (3)	0.5638 (2)	0.0401 (11)	
C11B	0.1546 (3)	−0.1297 (3)	0.5778 (2)	0.0399 (10)	
C13B	0.0824 (3)	−0.0154 (3)	0.6397 (3)	0.0407 (11)	
C14B	−0.0046 (2)	−0.0469 (3)	0.6264 (2)	0.0406 (11)	
C15B	0.2225 (4)	−0.1969 (4)	0.4448 (3)	0.0794 (17)	
H15A	0.2190	−0.2411	0.4127	0.119*	
H15B	0.2734	−0.1577	0.4355	0.119*	
H15C	0.1641	−0.1650	0.4460	0.119*	
C16B	0.3307 (3)	−0.2941 (4)	0.5059 (3)	0.0820 (19)	
H16A	0.3447	−0.3180	0.5468	0.123*	
H16B	0.3818	−0.2573	0.4925	0.123*	
H16C	0.3226	−0.3408	0.4760	0.123*	
C17B	−0.0042 (3)	0.1733 (3)	0.6459 (3)	0.0672 (15)	
H17A	−0.0608	0.2055	0.6547	0.101*	
H17B	−0.0127	0.1394	0.6081	0.101*	
H17C	0.0475	0.2131	0.6401	0.101*	
C18B	0.0376 (4)	0.1652 (4)	0.7612 (3)	0.0847 (19)	
H18A	−0.0162	0.2009	0.7711	0.127*	
H18B	0.0917	0.2018	0.7544	0.127*	
H18C	0.0499	0.1262	0.7959	0.127*	
C19B	−0.0573 (3)	−0.0748 (3)	0.5145 (2)	0.0448 (12)	
C20B	−0.0120 (3)	−0.0035 (3)	0.4882 (2)	0.0424 (12)	
H20A	0.0406	0.0195	0.5087	0.051*	
C21B	−0.0421 (3)	0.0343 (3)	0.4328 (2)	0.0466 (11)	
C22B	−0.1219 (3)	0.0023 (3)	0.4027 (3)	0.0522 (14)	
C23B	−0.1665 (3)	−0.0674 (4)	0.4282 (3)	0.083 (2)	
H23A	−0.2191	−0.0906	0.4079	0.100*	
C24B	−0.1351 (3)	−0.1051 (4)	0.4845 (3)	0.0768 (18)	
H24A	−0.1682	−0.1518	0.5017	0.092*	
C26B	0.0708 (3)	0.1487 (4)	0.4394 (3)	0.0827 (19)	
H26A	0.0927	0.1984	0.4159	0.124*	
H26B	0.1224	0.1091	0.4463	0.124*	
H26C	0.0461	0.1676	0.4794	0.124*	
C28B	−0.2427 (3)	0.0244 (3)	0.3247 (3)	0.0665 (15)	
H28A	−0.2557	0.0605	0.2887	0.100*	
H28B	−0.2880	0.0362	0.3574	0.100*	
H28C	−0.2466	−0.0360	0.3127	0.100*	

### Atomic displacement parameters (Å^2^)

**Table:**

	*U*^11^	*U*^22^	*U*^33^	*U*^12^	*U*^13^	*U*^23^
O1A	0.0224 (15)	0.062 (2)	0.080 (3)	0.0021 (15)	0.0027 (15)	0.0113 (19)
O8A	0.0491 (19)	0.047 (2)	0.103 (3)	−0.0204 (15)	0.0055 (18)	−0.010 (2)
O27A	0.056 (2)	0.093 (3)	0.056 (2)	−0.0114 (19)	−0.0208 (18)	0.008 (2)
N12A	0.0218 (16)	0.045 (2)	0.076 (3)	−0.0030 (15)	0.0028 (17)	−0.020 (2)
C1A	0.035 (3)	0.038 (3)	0.055 (3)	0.0047 (18)	0.001 (2)	0.008 (2)
C2A	0.040 (3)	0.055 (3)	0.071 (4)	0.005 (2)	0.012 (2)	−0.005 (3)
C3A	0.042 (2)	0.043 (2)	0.052 (3)	0.010 (2)	0.003 (2)	−0.005 (2)
C4A	0.030 (2)	0.046 (3)	0.059 (3)	0.0029 (19)	−0.005 (2)	−0.013 (2)
C5A	0.036 (2)	0.047 (3)	0.050 (3)	0.0037 (19)	−0.002 (2)	−0.015 (2)
C6A	0.041 (2)	0.039 (3)	0.043 (3)	0.0016 (19)	−0.001 (2)	−0.003 (2)
C7A	0.049 (3)	0.037 (2)	0.054 (3)	−0.001 (2)	−0.002 (2)	0.004 (2)
C8A	0.046 (3)	0.042 (3)	0.043 (3)	−0.008 (2)	−0.003 (2)	0.004 (2)
C9A	0.024 (2)	0.037 (2)	0.061 (3)	−0.0044 (17)	−0.0048 (19)	0.003 (2)
C10A	0.023 (2)	0.034 (2)	0.054 (3)	0.0009 (18)	−0.0026 (18)	0.002 (2)
C11A	0.032 (2)	0.040 (2)	0.034 (3)	0.0054 (16)	−0.0045 (19)	−0.005 (2)
C13A	0.028 (2)	0.041 (2)	0.046 (3)	−0.0004 (17)	0.0014 (18)	−0.005 (2)
C14A	0.028 (2)	0.036 (2)	0.045 (3)	−0.0037 (17)	−0.0025 (18)	−0.008 (2)
C15A	0.056 (3)	0.053 (3)	0.096 (4)	−0.001 (3)	−0.010 (3)	0.021 (3)
C16A	0.072 (4)	0.058 (4)	0.084 (4)	0.017 (3)	0.003 (3)	−0.021 (3)
C17A	0.068 (3)	0.060 (3)	0.052 (3)	0.005 (3)	0.002 (2)	0.004 (3)
C18A	0.055 (3)	0.052 (3)	0.070 (3)	0.015 (2)	0.003 (3)	−0.017 (3)
C19A	0.029 (2)	0.039 (2)	0.053 (3)	−0.0024 (16)	−0.0120 (18)	−0.004 (2)
C22A	0.037 (2)	0.062 (3)	0.050 (3)	−0.0014 (18)	−0.011 (2)	0.000 (2)
O25A	0.068 (5)	0.093 (6)	0.060 (5)	−0.036 (5)	−0.011 (4)	0.020 (4)
C20A	0.033 (4)	0.051 (6)	0.046 (5)	−0.016 (4)	−0.004 (3)	−0.010 (3)
C21A	0.033 (4)	0.056 (5)	0.046 (4)	−0.006 (3)	0.007 (3)	0.001 (3)
C23A	0.050 (7)	0.063 (7)	0.081 (7)	−0.012 (5)	−0.036 (6)	0.008 (6)
C24A	0.041 (3)	0.046 (5)	0.078 (4)	−0.012 (3)	−0.027 (3)	0.011 (4)
C26A	0.055 (9)	0.071 (8)	0.108 (13)	−0.016 (6)	0.005 (7)	−0.005 (8)
C19C	0.029 (2)	0.039 (2)	0.053 (3)	−0.0024 (16)	−0.0120 (18)	−0.004 (2)
C22C	0.037 (2)	0.062 (3)	0.050 (3)	−0.0014 (18)	−0.011 (2)	0.000 (2)
O25C	0.093 (11)	0.127 (17)	0.056 (9)	−0.057 (12)	−0.011 (9)	0.028 (11)
C20C	0.027 (7)	0.046 (10)	0.056 (9)	0.004 (6)	−0.010 (5)	0.006 (7)
C21C	0.048 (8)	0.080 (13)	0.039 (9)	−0.022 (8)	−0.010 (6)	0.004 (7)
C23C	0.029 (9)	0.077 (15)	0.081 (12)	−0.003 (7)	−0.022 (7)	0.020 (10)
C24C	0.041 (3)	0.046 (5)	0.078 (4)	−0.012 (3)	−0.027 (3)	0.011 (4)
C26C	0.054 (15)	0.11 (3)	0.083 (17)	−0.05 (2)	0.001 (15)	0.02 (2)
C28A	0.048 (3)	0.103 (5)	0.066 (4)	0.004 (3)	−0.030 (3)	−0.005 (3)
O1B	0.063 (2)	0.050 (2)	0.130 (4)	−0.0236 (19)	−0.002 (2)	−0.003 (2)
O8B	0.0238 (16)	0.066 (2)	0.131 (4)	0.0006 (16)	0.0040 (18)	0.018 (2)
O25B	0.080 (2)	0.080 (3)	0.070 (3)	−0.046 (2)	−0.0272 (19)	0.031 (2)
O27B	0.0521 (19)	0.074 (2)	0.055 (2)	−0.0110 (17)	−0.0196 (16)	0.0152 (19)
N12B	0.0212 (17)	0.054 (2)	0.062 (3)	−0.0031 (16)	−0.0006 (16)	−0.004 (2)
C1B	0.060 (3)	0.043 (3)	0.059 (3)	−0.013 (2)	0.002 (2)	0.007 (3)
C2B	0.060 (3)	0.044 (3)	0.072 (4)	0.008 (2)	0.008 (3)	0.007 (3)
C3B	0.047 (3)	0.052 (3)	0.053 (3)	−0.001 (2)	0.009 (2)	0.005 (3)
C4B	0.037 (2)	0.055 (3)	0.058 (3)	0.004 (2)	0.010 (2)	0.005 (3)
C5B	0.039 (2)	0.062 (3)	0.058 (3)	0.010 (2)	−0.006 (2)	−0.006 (3)
C6B	0.036 (2)	0.058 (3)	0.066 (4)	0.010 (2)	0.004 (2)	−0.001 (3)
C7B	0.049 (3)	0.055 (3)	0.082 (4)	0.013 (2)	0.017 (3)	0.012 (3)
C8B	0.028 (3)	0.050 (3)	0.069 (4)	0.006 (2)	0.004 (2)	0.021 (3)
C9B	0.025 (2)	0.038 (2)	0.064 (3)	−0.0089 (18)	−0.001 (2)	0.017 (2)
C10B	0.032 (2)	0.042 (3)	0.047 (3)	−0.0053 (18)	0.0007 (19)	0.013 (2)
C11B	0.035 (2)	0.040 (2)	0.044 (3)	−0.0010 (19)	0.0000 (19)	0.000 (2)
C13B	0.026 (2)	0.043 (3)	0.054 (3)	0.0071 (18)	0.001 (2)	0.005 (2)
C14B	0.024 (2)	0.042 (3)	0.056 (3)	0.0056 (18)	−0.001 (2)	0.014 (2)
C15B	0.098 (4)	0.079 (4)	0.061 (4)	−0.008 (3)	0.018 (3)	0.012 (3)
C16B	0.063 (3)	0.084 (5)	0.100 (5)	0.020 (3)	0.021 (3)	−0.008 (4)
C17B	0.052 (3)	0.048 (3)	0.102 (4)	−0.002 (2)	0.001 (3)	0.018 (3)
C18B	0.083 (4)	0.082 (5)	0.089 (4)	0.019 (3)	0.002 (3)	−0.031 (4)
C19B	0.030 (2)	0.042 (3)	0.062 (3)	−0.0089 (19)	−0.005 (2)	0.012 (2)
C20B	0.033 (2)	0.047 (3)	0.048 (3)	−0.0096 (18)	−0.012 (2)	0.012 (2)
C21B	0.041 (2)	0.049 (3)	0.050 (3)	−0.011 (2)	−0.006 (2)	0.010 (3)
C22B	0.050 (3)	0.056 (3)	0.051 (3)	−0.010 (2)	−0.019 (3)	0.009 (3)
C23B	0.065 (3)	0.088 (4)	0.097 (5)	−0.039 (3)	−0.048 (3)	0.036 (4)
C24B	0.066 (3)	0.072 (4)	0.093 (4)	−0.039 (3)	−0.037 (3)	0.044 (3)
C26B	0.070 (3)	0.087 (4)	0.091 (4)	−0.054 (3)	−0.029 (3)	0.035 (4)
C28B	0.052 (3)	0.086 (4)	0.062 (4)	−0.004 (3)	−0.021 (3)	0.006 (3)

### Geometric parameters (Å, º)

**Table:**

O1A—C1A	1.227 (5)	C26C—H26H	0.9600
O8A—C8A	1.225 (4)	C26C—H26I	0.9600
O27A—C22A	1.357 (6)	C28A—H28D	0.9600
O27A—C28A	1.425 (5)	C28A—H28E	0.9600
N12A—C13A	1.372 (5)	C28A—H28F	0.9600
N12A—C11A	1.377 (5)	O1B—C1B	1.233 (5)
N12A—H12A	0.8600	O8B—C8B	1.238 (5)
C1A—C10A	1.441 (6)	O25B—C21B	1.373 (5)
C1A—C2A	1.497 (7)	O25B—C26B	1.417 (5)
C2A—C3A	1.532 (6)	O27B—C22B	1.368 (6)
C2A—H2AA	0.9700	O27B—C28B	1.443 (5)
C2A—H2AB	0.9700	N12B—C11B	1.378 (5)
C3A—C4A	1.523 (5)	N12B—C13B	1.382 (5)
C3A—C16A	1.525 (6)	N12B—H12B	0.8600
C3A—C15A	1.531 (6)	C1B—C10B	1.453 (6)
C4A—C11A	1.487 (6)	C1B—C2B	1.511 (6)
C4A—H4AA	0.9700	C2B—C3B	1.525 (6)
C4A—H4AB	0.9700	C2B—H2BA	0.9700
C5A—C13A	1.501 (5)	C2B—H2BB	0.9700
C5A—C6A	1.527 (5)	C3B—C4B	1.532 (6)
C5A—H5AA	0.9700	C3B—C16B	1.532 (6)
C5A—H5AB	0.9700	C3B—C15B	1.537 (6)
C6A—C7A	1.518 (6)	C4B—C11B	1.510 (5)
C6A—C17A	1.530 (6)	C4B—H4BA	0.9700
C6A—C18A	1.536 (5)	C4B—H4BB	0.9700
C7A—C8A	1.512 (6)	C5B—C13B	1.474 (6)
C7A—H7AA	0.9700	C5B—C6B	1.533 (5)
C7A—H7AB	0.9700	C5B—H5BA	0.9700
C8A—C14A	1.444 (5)	C5B—H5BB	0.9700
C9A—C19A	1.518 (5)	C6B—C18B	1.528 (7)
C9A—C14A	1.519 (5)	C6B—C17B	1.530 (6)
C9A—C10A	1.520 (6)	C6B—C7B	1.531 (6)
C9A—H9AA	0.9800	C7B—C8B	1.489 (7)
C10A—C11A	1.351 (5)	C7B—H7BA	0.9700
C13A—C14A	1.348 (5)	C7B—H7BB	0.9700
C15A—H15D	0.9600	C8B—C14B	1.446 (6)
C15A—H15E	0.9600	C9B—C14B	1.499 (6)
C15A—H15F	0.9600	C9B—C19B	1.516 (6)
C16A—H16D	0.9600	C9B—C10B	1.520 (5)
C16A—H16E	0.9600	C9B—H9BA	0.9800
C16A—H16F	0.9600	C10B—C11B	1.348 (5)
C17A—H17D	0.9600	C13B—C14B	1.352 (5)
C17A—H17E	0.9600	C15B—H15A	0.9600
C17A—H17F	0.9600	C15B—H15B	0.9600
C18A—H18D	0.9600	C15B—H15C	0.9600
C18A—H18E	0.9600	C16B—H16A	0.9600
C18A—H18F	0.9600	C16B—H16B	0.9600
C19A—C24A	1.376 (5)	C16B—H16C	0.9600
C19A—C20A	1.394 (7)	C17B—H17A	0.9600
C22A—C23A	1.379 (7)	C17B—H17B	0.9600
C22A—C21A	1.404 (7)	C17B—H17C	0.9600
O25A—C21A	1.366 (7)	C18B—H18A	0.9600
O25A—C26A	1.380 (7)	C18B—H18B	0.9600
C20A—C21A	1.356 (8)	C18B—H18C	0.9600
C20A—H20B	0.9300	C19B—C24B	1.353 (5)
C23A—C24A	1.394 (7)	C19B—C20B	1.383 (6)
C23A—H23B	0.9300	C20B—C21B	1.373 (6)
C24A—H24B	0.9300	C20B—H20A	0.9300
C26A—H26D	0.9600	C21B—C22B	1.387 (6)
C26A—H26E	0.9600	C22B—C23B	1.353 (6)
C26A—H26F	0.9600	C23B—C24B	1.393 (6)
O25C—C21C	1.366 (7)	C23B—H23A	0.9300
O25C—C26C	1.380 (7)	C24B—H24A	0.9300
C20C—C21C	1.356 (8)	C26B—H26A	0.9600
C20C—H20C	0.9300	C26B—H26B	0.9600
C23C—C24C	1.394 (7)	C26B—H26C	0.9600
C23C—H23C	0.9300	C28B—H28A	0.9600
C24C—H24C	0.9300	C28B—H28B	0.9600
C26C—H26G	0.9600	C28B—H28C	0.9600
			
C22A—O27A—C28A	117.3 (4)	H28D—C28A—H28F	109.5
C13A—N12A—C11A	121.3 (3)	H28E—C28A—H28F	109.5
C13A—N12A—H12A	119.4	C21B—O25B—C26B	116.7 (4)
C11A—N12A—H12A	119.4	C22B—O27B—C28B	117.0 (4)
O1A—C1A—C10A	121.7 (4)	C11B—N12B—C13B	121.8 (3)
O1A—C1A—C2A	120.4 (4)	C11B—N12B—H12B	119.1
C10A—C1A—C2A	117.9 (4)	C13B—N12B—H12B	119.1
C1A—C2A—C3A	113.8 (4)	O1B—C1B—C10B	120.7 (4)
C1A—C2A—H2AA	108.8	O1B—C1B—C2B	121.0 (5)
C3A—C2A—H2AA	108.8	C10B—C1B—C2B	118.2 (4)
C1A—C2A—H2AB	108.8	C1B—C2B—C3B	113.8 (4)
C3A—C2A—H2AB	108.8	C1B—C2B—H2BA	108.8
H2AA—C2A—H2AB	107.7	C3B—C2B—H2BA	108.8
C4A—C3A—C16A	109.1 (3)	C1B—C2B—H2BB	108.8
C4A—C3A—C15A	111.0 (4)	C3B—C2B—H2BB	108.8
C16A—C3A—C15A	109.4 (4)	H2BA—C2B—H2BB	107.7
C4A—C3A—C2A	107.4 (4)	C2B—C3B—C4B	109.0 (4)
C16A—C3A—C2A	110.2 (4)	C2B—C3B—C16B	110.0 (4)
C15A—C3A—C2A	109.8 (4)	C4B—C3B—C16B	109.9 (4)
C11A—C4A—C3A	112.9 (3)	C2B—C3B—C15B	108.5 (4)
C11A—C4A—H4AA	109.0	C4B—C3B—C15B	110.8 (4)
C3A—C4A—H4AA	109.0	C16B—C3B—C15B	108.7 (4)
C11A—C4A—H4AB	109.0	C11B—C4B—C3B	112.5 (4)
C3A—C4A—H4AB	109.0	C11B—C4B—H4BA	109.1
H4AA—C4A—H4AB	107.8	C3B—C4B—H4BA	109.1
C13A—C5A—C6A	112.3 (3)	C11B—C4B—H4BB	109.1
C13A—C5A—H5AA	109.1	C3B—C4B—H4BB	109.1
C6A—C5A—H5AA	109.1	H4BA—C4B—H4BB	107.8
C13A—C5A—H5AB	109.1	C13B—C5B—C6B	114.1 (4)
C6A—C5A—H5AB	109.1	C13B—C5B—H5BA	108.7
H5AA—C5A—H5AB	107.9	C6B—C5B—H5BA	108.7
C7A—C6A—C5A	108.0 (4)	C13B—C5B—H5BB	108.7
C7A—C6A—C17A	110.4 (3)	C6B—C5B—H5BB	108.7
C5A—C6A—C17A	110.5 (4)	H5BA—C5B—H5BB	107.6
C7A—C6A—C18A	108.9 (4)	C18B—C6B—C17B	110.3 (4)
C5A—C6A—C18A	108.9 (3)	C18B—C6B—C7B	110.3 (4)
C17A—C6A—C18A	110.1 (4)	C17B—C6B—C7B	108.8 (4)
C8A—C7A—C6A	114.3 (3)	C18B—C6B—C5B	109.5 (4)
C8A—C7A—H7AA	108.7	C17B—C6B—C5B	111.2 (4)
C6A—C7A—H7AA	108.7	C7B—C6B—C5B	106.6 (4)
C8A—C7A—H7AB	108.7	C8B—C7B—C6B	114.6 (4)
C6A—C7A—H7AB	108.7	C8B—C7B—H7BA	108.6
H7AA—C7A—H7AB	107.6	C6B—C7B—H7BA	108.6
O8A—C8A—C14A	121.7 (4)	C8B—C7B—H7BB	108.6
O8A—C8A—C7A	120.4 (4)	C6B—C7B—H7BB	108.6
C14A—C8A—C7A	117.7 (3)	H7BA—C7B—H7BB	107.6
C19A—C9A—C14A	111.1 (3)	O8B—C8B—C14B	120.5 (5)
C19A—C9A—C10A	112.0 (3)	O8B—C8B—C7B	121.4 (4)
C14A—C9A—C10A	109.2 (3)	C14B—C8B—C7B	118.1 (4)
C19A—C9A—H9AA	108.1	C14B—C9B—C19B	113.1 (4)
C14A—C9A—H9AA	108.1	C14B—C9B—C10B	109.9 (3)
C10A—C9A—H9AA	108.1	C19B—C9B—C10B	110.4 (4)
C11A—C10A—C1A	119.4 (4)	C14B—C9B—H9BA	107.8
C11A—C10A—C9A	121.7 (4)	C19B—C9B—H9BA	107.8
C1A—C10A—C9A	118.9 (3)	C10B—C9B—H9BA	107.8
C10A—C11A—N12A	120.0 (4)	C11B—C10B—C1B	119.6 (4)
C10A—C11A—C4A	124.5 (4)	C11B—C10B—C9B	120.8 (4)
N12A—C11A—C4A	115.5 (3)	C1B—C10B—C9B	119.6 (4)
C14A—C13A—N12A	121.0 (3)	C10B—C11B—N12B	120.4 (4)
C14A—C13A—C5A	123.7 (4)	C10B—C11B—C4B	124.5 (4)
N12A—C13A—C5A	115.2 (3)	N12B—C11B—C4B	115.1 (3)
C13A—C14A—C8A	120.1 (3)	C14B—C13B—N12B	119.5 (4)
C13A—C14A—C9A	120.9 (4)	C14B—C13B—C5B	124.5 (4)
C8A—C14A—C9A	119.0 (3)	N12B—C13B—C5B	115.9 (3)
C3A—C15A—H15D	109.5	C13B—C14B—C8B	118.9 (4)
C3A—C15A—H15E	109.5	C13B—C14B—C9B	122.0 (4)
H15D—C15A—H15E	109.5	C8B—C14B—C9B	119.0 (4)
C3A—C15A—H15F	109.5	C3B—C15B—H15A	109.5
H15D—C15A—H15F	109.5	C3B—C15B—H15B	109.5
H15E—C15A—H15F	109.5	H15A—C15B—H15B	109.5
C3A—C16A—H16D	109.5	C3B—C15B—H15C	109.5
C3A—C16A—H16E	109.5	H15A—C15B—H15C	109.5
H16D—C16A—H16E	109.5	H15B—C15B—H15C	109.5
C3A—C16A—H16F	109.5	C3B—C16B—H16A	109.5
H16D—C16A—H16F	109.5	C3B—C16B—H16B	109.5
H16E—C16A—H16F	109.5	H16A—C16B—H16B	109.5
C6A—C17A—H17D	109.5	C3B—C16B—H16C	109.5
C6A—C17A—H17E	109.5	H16A—C16B—H16C	109.5
H17D—C17A—H17E	109.5	H16B—C16B—H16C	109.5
C6A—C17A—H17F	109.5	C6B—C17B—H17A	109.5
H17D—C17A—H17F	109.5	C6B—C17B—H17B	109.5
H17E—C17A—H17F	109.5	H17A—C17B—H17B	109.5
C6A—C18A—H18D	109.5	C6B—C17B—H17C	109.5
C6A—C18A—H18E	109.5	H17A—C17B—H17C	109.5
H18D—C18A—H18E	109.5	H17B—C17B—H17C	109.5
C6A—C18A—H18F	109.5	C6B—C18B—H18A	109.5
H18D—C18A—H18F	109.5	C6B—C18B—H18B	109.5
H18E—C18A—H18F	109.5	H18A—C18B—H18B	109.5
C24A—C19A—C20A	118.2 (4)	C6B—C18B—H18C	109.5
C24A—C19A—C9A	119.6 (4)	H18A—C18B—H18C	109.5
C20A—C19A—C9A	122.1 (4)	H18B—C18B—H18C	109.5
O27A—C22A—C23A	125.6 (5)	C24B—C19B—C20B	117.4 (4)
O27A—C22A—C21A	117.0 (5)	C24B—C19B—C9B	122.9 (4)
C23A—C22A—C21A	117.4 (5)	C20B—C19B—C9B	119.7 (4)
C21A—O25A—C26A	119.4 (6)	C21B—C20B—C19B	122.1 (4)
C21A—C20A—C19A	122.0 (5)	C21B—C20B—H20A	119.0
C21A—C20A—H20B	119.0	C19B—C20B—H20A	119.0
C19A—C20A—H20B	119.0	C20B—C21B—O25B	125.0 (4)
C20A—C21A—O25A	124.8 (5)	C20B—C21B—C22B	119.7 (4)
C20A—C21A—C22A	120.6 (5)	O25B—C21B—C22B	115.2 (4)
O25A—C21A—C22A	114.6 (5)	C23B—C22B—O27B	124.5 (4)
C22A—C23A—C24A	122.0 (5)	C23B—C22B—C21B	118.5 (5)
C22A—C23A—H23B	119.0	O27B—C22B—C21B	117.1 (4)
C24A—C23A—H23B	119.0	C22B—C23B—C24B	121.2 (4)
C19A—C24A—C23A	119.8 (5)	C22B—C23B—H23A	119.4
C19A—C24A—H24B	120.1	C24B—C23B—H23A	119.4
C23A—C24A—H24B	120.1	C19B—C24B—C23B	121.2 (4)
C21C—O25C—C26C	119.3 (6)	C19B—C24B—H24A	119.4
C21C—C20C—H20C	119.0	C23B—C24B—H24A	119.4
C20C—C21C—O25C	124.7 (5)	O25B—C26B—H26A	109.5
C24C—C23C—H23C	119.1	O25B—C26B—H26B	109.5
C23C—C24C—H24C	120.1	H26A—C26B—H26B	109.5
O25C—C26C—H26G	109.5	O25B—C26B—H26C	109.5
O25C—C26C—H26H	109.5	H26A—C26B—H26C	109.5
H26G—C26C—H26H	109.5	H26B—C26B—H26C	109.5
O25C—C26C—H26I	109.5	O27B—C28B—H28A	109.5
H26G—C26C—H26I	109.5	O27B—C28B—H28B	109.5
H26H—C26C—H26I	109.5	H28A—C28B—H28B	109.5
O27A—C28A—H28D	109.5	O27B—C28B—H28C	109.5
O27A—C28A—H28E	109.5	H28A—C28B—H28C	109.5
H28D—C28A—H28E	109.5	H28B—C28B—H28C	109.5
O27A—C28A—H28F	109.5		
			
O1A—C1A—C2A—C3A	148.7 (4)	O1B—C1B—C2B—C3B	151.5 (5)
C10A—C1A—C2A—C3A	−33.4 (6)	C10B—C1B—C2B—C3B	−31.4 (6)
C1A—C2A—C3A—C4A	54.9 (5)	C1B—C2B—C3B—C4B	53.3 (6)
C1A—C2A—C3A—C16A	173.6 (4)	C1B—C2B—C3B—C16B	173.8 (4)
C1A—C2A—C3A—C15A	−65.9 (5)	C1B—C2B—C3B—C15B	−67.4 (6)
C16A—C3A—C4A—C11A	−167.4 (4)	C2B—C3B—C4B—C11B	−46.6 (5)
C15A—C3A—C4A—C11A	72.0 (5)	C16B—C3B—C4B—C11B	−167.2 (4)
C2A—C3A—C4A—C11A	−48.1 (5)	C15B—C3B—C4B—C11B	72.6 (5)
C13A—C5A—C6A—C7A	49.7 (5)	C13B—C5B—C6B—C18B	166.2 (5)
C13A—C5A—C6A—C17A	−71.2 (4)	C13B—C5B—C6B—C17B	−71.7 (5)
C13A—C5A—C6A—C18A	167.8 (4)	C13B—C5B—C6B—C7B	46.8 (6)
C5A—C6A—C7A—C8A	−53.4 (5)	C18B—C6B—C7B—C8B	−172.3 (4)
C17A—C6A—C7A—C8A	67.5 (5)	C17B—C6B—C7B—C8B	66.5 (5)
C18A—C6A—C7A—C8A	−171.5 (4)	C5B—C6B—C7B—C8B	−53.5 (5)
C6A—C7A—C8A—O8A	−156.3 (4)	C6B—C7B—C8B—O8B	−146.9 (4)
C6A—C7A—C8A—C14A	28.0 (6)	C6B—C7B—C8B—C14B	35.2 (6)
O1A—C1A—C10A—C11A	−179.4 (4)	O1B—C1B—C10B—C11B	178.2 (4)
C2A—C1A—C10A—C11A	2.7 (6)	C2B—C1B—C10B—C11B	1.1 (7)
O1A—C1A—C10A—C9A	−0.3 (7)	O1B—C1B—C10B—C9B	−3.2 (7)
C2A—C1A—C10A—C9A	−178.2 (4)	C2B—C1B—C10B—C9B	179.7 (4)
C19A—C9A—C10A—C11A	100.3 (4)	C14B—C9B—C10B—C11B	−24.2 (5)
C14A—C9A—C10A—C11A	−23.3 (6)	C19B—C9B—C10B—C11B	101.1 (5)
C19A—C9A—C10A—C1A	−78.8 (5)	C14B—C9B—C10B—C1B	157.2 (4)
C14A—C9A—C10A—C1A	157.7 (4)	C19B—C9B—C10B—C1B	−77.5 (5)
C1A—C10A—C11A—N12A	−174.4 (4)	C1B—C10B—C11B—N12B	−172.4 (4)
C9A—C10A—C11A—N12A	6.6 (6)	C9B—C10B—C11B—N12B	9.0 (6)
C1A—C10A—C11A—C4A	3.5 (7)	C1B—C10B—C11B—C4B	5.0 (7)
C9A—C10A—C11A—C4A	−175.6 (4)	C9B—C10B—C11B—C4B	−173.7 (4)
C13A—N12A—C11A—C10A	12.7 (7)	C13B—N12B—C11B—C10B	10.4 (7)
C13A—N12A—C11A—C4A	−165.4 (4)	C13B—N12B—C11B—C4B	−167.2 (4)
C3A—C4A—C11A—C10A	21.3 (6)	C3B—C4B—C11B—C10B	19.3 (6)
C3A—C4A—C11A—N12A	−160.8 (4)	C3B—C4B—C11B—N12B	−163.2 (4)
C11A—N12A—C13A—C14A	−11.7 (7)	C11B—N12B—C13B—C14B	−11.4 (7)
C11A—N12A—C13A—C5A	165.7 (4)	C11B—N12B—C13B—C5B	164.6 (4)
C6A—C5A—C13A—C14A	−22.1 (6)	C6B—C5B—C13B—C14B	−23.1 (7)
C6A—C5A—C13A—N12A	160.6 (4)	C6B—C5B—C13B—N12B	161.2 (4)
N12A—C13A—C14A—C8A	171.2 (4)	N12B—C13B—C14B—C8B	176.4 (4)
C5A—C13A—C14A—C8A	−6.0 (6)	C5B—C13B—C14B—C8B	0.8 (7)
N12A—C13A—C14A—C9A	−8.4 (6)	N12B—C13B—C14B—C9B	−7.3 (6)
C5A—C13A—C14A—C9A	174.4 (4)	C5B—C13B—C14B—C9B	177.0 (4)
O8A—C8A—C14A—C13A	−172.4 (4)	O8B—C8B—C14B—C13B	175.3 (4)
C7A—C8A—C14A—C13A	3.2 (6)	C7B—C8B—C14B—C13B	−6.7 (6)
O8A—C8A—C14A—C9A	7.2 (6)	O8B—C8B—C14B—C9B	−1.0 (6)
C7A—C8A—C14A—C9A	−177.2 (4)	C7B—C8B—C14B—C9B	176.9 (4)
C19A—C9A—C14A—C13A	−100.0 (4)	C19B—C9B—C14B—C13B	−100.3 (4)
C10A—C9A—C14A—C13A	24.1 (5)	C10B—C9B—C14B—C13B	23.5 (6)
C19A—C9A—C14A—C8A	80.4 (5)	C19B—C9B—C14B—C8B	75.9 (5)
C10A—C9A—C14A—C8A	−155.5 (4)	C10B—C9B—C14B—C8B	−160.3 (4)
C14A—C9A—C19A—C24A	−100.4 (6)	C14B—C9B—C19B—C24B	−128.4 (5)
C10A—C9A—C19A—C24A	137.1 (6)	C10B—C9B—C19B—C24B	108.1 (5)
C14A—C9A—C19A—C20A	77.3 (6)	C14B—C9B—C19B—C20B	49.7 (5)
C10A—C9A—C19A—C20A	−45.1 (6)	C10B—C9B—C19B—C20B	−73.9 (5)
C28A—O27A—C22A—C23A	−16.7 (7)	C24B—C19B—C20B—C21B	−1.9 (7)
C28A—O27A—C22A—C21A	162.8 (6)	C9B—C19B—C20B—C21B	180.0 (4)
C24A—C19A—C20A—C21A	0.00 (10)	C19B—C20B—C21B—O25B	179.1 (5)
C9A—C19A—C20A—C21A	−177.8 (3)	C19B—C20B—C21B—C22B	1.8 (8)
C19A—C20A—C21A—O25A	180.00 (6)	C26B—O25B—C21B—C20B	−7.2 (7)
C19A—C20A—C21A—C22A	0.00 (11)	C26B—O25B—C21B—C22B	170.1 (5)
C26A—O25A—C21A—C20A	−0.4 (17)	C28B—O27B—C22B—C23B	15.7 (8)
C26A—O25A—C21A—C22A	179.6 (17)	C28B—O27B—C22B—C21B	−165.6 (5)
O27A—C22A—C21A—C20A	−179.5 (4)	C20B—C21B—C22B—C23B	−1.7 (8)
C23A—C22A—C21A—C20A	0.00 (9)	O25B—C21B—C22B—C23B	−179.2 (5)
O27A—C22A—C21A—O25A	0.5 (4)	C20B—C21B—C22B—O27B	179.5 (4)
C23A—C22A—C21A—O25A	180.00 (5)	O25B—C21B—C22B—O27B	2.0 (7)
O27A—C22A—C23A—C24A	179.5 (4)	O27B—C22B—C23B—C24B	−179.5 (5)
C21A—C22A—C23A—C24A	0.00 (9)	C21B—C22B—C23B—C24B	1.7 (9)
C20A—C19A—C24A—C23A	0.00 (9)	C20B—C19B—C24B—C23B	1.9 (8)
C9A—C19A—C24A—C23A	177.8 (3)	C9B—C19B—C24B—C23B	180.0 (5)
C22A—C23A—C24A—C19A	0.00 (11)	C22B—C23B—C24B—C19B	−1.9 (10)
C26C—O25C—C21C—C20C	20 (4)		

### Hydrogen-bond geometry (Å, º)

**Table:**

*D*—H···*A*	*D*—H	H···*A*	*D*···*A*	*D*—H···*A*
N12*A*—H12*A*···O1*A*^i^	0.86	2.07	2.846 (4)	150
N12*B*—H12*B*···O8*B*^ii^	0.86	2.05	2.885 (4)	165
C2*A*—H2*AA*···O1*B*^ii^	0.97	2.45	3.400 (6)	166
C16*A*—H16*D*···O25*A*^iii^	0.96	2.61	3.477 (12)	150
C7*A*—H7*AB*···O27*B*^iv^	0.97	2.47	3.432 (6)	169
C7*B*—H7*BB*···O8*A*^i^	0.97	2.57	3.507 (5)	162

Symmetry codes: (i) *x*−1/2, −*y*+1, *z*; (ii) *x*+1/2, −*y*, *z*; (iii) −*x*+1/2, *y*, *z*−1/2; (iv) −*x*, −*y*+1, *z*+1/2.
